# The Discussion Goes on: What Is the Role of *Euryarchaeota* in Humans?

**DOI:** 10.1155/2010/967271

**Published:** 2010-12-30

**Authors:** H.-P. Horz, G. Conrads

**Affiliations:** Division of Oral Microbiology and Immunology, Department of Operative and Preventive Dentistry & Periodontology, and Department of Medical Microbiology, RWTH Aachen University Hospital, 52057 Aachen, Germany

## Abstract

The human body (primarily the intestinal tract, the oral cavity, and the skin) harbours approximately 1,000 different bacterial species. However, the number of archaeal species known to colonize man seems to be confined to a handful of organisms within the class *Euryarchaeota* (including *Methanobrevibacter smithii*, *M. oralis*, and *Methanosphaera stadtmanae*). In contrast to this conspicuously low diversity of *Archaea* in humans their unique physiology in conjunction with the growing number of reports regarding their occurrence at sites of infection has made this issue an emerging field of study. While previous review articles in recent years have addressed the putative role of particularly methanogenic archaea for human health and disease, this paper compiles novel experimental data that have been reported since then. The aim of this paper is to inspire the scientific community of “*Archaea* experts” for those unique archaeal organisms that have successfully participated in the human-microbe coevolution.

## 1. Introduction

A striking feature of the human microbiota is the conspicuous imbalance of species diversity between bacteria and archaea. So far, over thousand distinct bacterial species or phylotypes have been recovered from the human intestinal tract, and more than 700 bacterial phylotypes have been identified in the human oral cavity (e.g., [1, 2]). Although most intestinal microbes initially enter through the oral cavity, both compartments share surprisingly few bacterial species [3, 4]. The underlying mechanisms that lead to the spatial separation of bacterial communities seem to apply also to human archaea; however, the striking difference is the extremely reduced diversity compared to bacteria. Only three distinct species within the group of *Euryarchaeota* have been regularly detected within the human body. Among these is the primary colonizer of the human gut system *Methanobrevibacter smithii* and the less frequently found species *Methanosphaera stadtmanae*, while in the oral cavity *M. oralis* is the predominating methanogenic species. Despite this low diversity and the generally lower abundance compared to human bacteria the unique physiology and energy metabolism of methanogens suggest that they may play a previously underestimated role for human health and disease. Previous reviews have already discussed the theoretical possibility that archaea might act as human pathogens (e.g., [5–7]). The current paper while not recapitulating these reviews compiles knowledge from most recent findings (arising from more than 20 additional studies, primarily from the last three years) that have substantially complemented our view of diversity and prevalence of euryarchaeota in humans as well as their association with health and disease.

## 2. How Can Archaea Affect Human Health?

Before discussing novel findings, it should be pointed out that there are two basic mechanisms under discussion by which methanogens theoretically could influence our health. First is through interspecies hydrogen transfer [6], a mutually beneficial, unidirectional process that plays a central role in the anaerobic fermentation of organic matter in natural environments. By ways of syntrophic interactions, this means that methanogens in humans might support the growth of fermenting bacteria, which themselves could be either true pathogens or at least opportunistic pathogens but also members of the physiological flora (so-called commensals), which influence our health in other indirect ways. The support of human pathogens is feasible at sites of infections, like periodontal pockets or infected tooth-root canals, where methanogens have been frequently identified. The support of a physiological flora is feasible in the human intestinal tract system. Here, even in the absence of an acute infection, the type of interactions, substrates, and end-products (i.e., the type of syntrophic partners) may nonetheless be decisive for the status of health (e.g., colon cancer or obesity, see later in this paper). Of course methanogens are not the only hydrogenotrophic groups as sulfate-reducers and acetogenic bacteria both of which are present in the human body potentially are also capable of analogous interactions (e.g., reviewed in [8]). Even if these three functional microbial guilds are alternatively involved in interspecies hydrogen transfer depending on environmental conditions and the individual ecological niche, they can—as a whole—be considered as keystone organisms that coregulate the activity of the remaining fermenting microbiota and as such are responsible for the overall microbial homeostasis. 

The second mechanism under discussion is the capability of methanogens to effectively transform heavy metals or metalloids into volatile methylated derivatives which are known to be more toxic than the original compounds [9]. Again, also this feature is shared with some bacteria, but interestingly, methanogens, isolated from the human gut, have been shown to possess a much higher potential for metal(loid) derivatization (e.g., bismuth, selenium, tellurium, and mercury) *in vitro* compared to bacterial gut isolates [10]. The immediate consequences of such transformation for human health have to be elucidated. However, as an example use of bismuth containing compounds in pharmaceutical products has been linked with poisoning during prolonged medical therapy with the consequences of renal failures and mental disorders as described by Michalke et al. [9]. It is likely that frequent exposure with such heavy metals through use of cosmetics or pharmaceutical products increases the possibility of its methylation by methanogens followed by increased toxicity. Whether or not methanogens in the oral cavity also have the potential or opportunity for such toxic transformations has not been investigated so far, but given the high number of foreign material used in dentistry this could be of great clinical importance as well. 

While the first mechanism mentioned above would constitute an indirect form of pathogenesis, the second mechanism would actually be a direct virulence factor. In any case, if one or both of those mechanisms have an affect on our health this would constitute a novel paradigm of microbial pathogenesis and could open new horizons for future research including the challenge of developing novel concepts for therapy of infectious diseases and/or human physiological disorders.

## 3. Methanogens in the Oral Cavity: What Is New?

*Methanobrevibacter oralis*, the major archaeal player in the oral cavity, has long been identified in periodontal pockets. Its proportional increase with severity of disease has meanwhile been confirmed by various groups. Whether or not interspecies hydrogen transfer is a driving force of this polymicrobial disease remains still unknown. However, our recent cross-sectional analysis of periodontal samples from more than one hundred patients based on selected marker genes (i.e., *mcrA*, *dsrAB*, and *fhs*) not only revealed the consistent presence of the three major hydrogenotrophic microbial groups (i.e., methanogens, dissimilatory sulfate reducers, and reductive acetogens) but also demonstrated negative interactions among these as well as an increase of their relative proportions with severity of the disease [11]. Given the varying microbial interactions and the overall heterogeneity of the plaque biofilm composition, both temporarily and among individuals, the observed pattern suggested that antagonistic interactions among H_2_-utilizers seem to prevail, with the dominance of methanogens and sulfate-reducers over acetogens being associated with a clinically more harmful situation for the host. 

Human archaea have become into another potentially compromising position as *M. oralis* was identified in a specific form of oral infections, namely, apical periodontitis, by our group recently [12]. Apical periodontitis is the result of the infection of a tooth's root canal, and it has long been known that this location—devoid of microbes in a healthy state—can only be invaded steadily by few distinct members of the oral microflora. Apparently these organisms, which are generally referred to as endodontic pathogens, have the properties necessary to invade tubules and to survive within the intratubular environment. Yet, quite naturally, one hesitates to apply this term also to *M. oralis* simply based on its presence in infected root canals. However, this aspect certainly warrants further investigation. Recent studies on US patients and Japanese patients have confirmed the presence of *M. oralis* in infected root canals [13, 14]. The latter study provided three additional important aspects worth notifying. First, the combined presence of archaea (i.e., *M. oralis*) and bacteria was associated with a significantly higher prevalence of clinical symptoms (e.g., pain) compared to the number of cases with sole presence of bacteria. Second, archaea were identified also in persisting or secondary endodontic infections, that is, in cases of failed root canal treatment, usually the result of microbes surviving the disinfection procedures. This is interesting, since we do not know how archaea respond to classical endodontic disinfectants such as sodium hypochloride or chlorhexidine. Lastly, Jiang et al. [14] identified archaea using an rRNA-based approach. This demonstrates that *M. oralis* was in fact present in a viable state in infected root canals and reduces speculations that the former DNA-based studies only detected the—in comparison to RNA—much more stable DNA released from dead or damaged archaeal cells. 

First data about the immunogenicity of *M. oralis* after oral infections has also been obtained most recently. Yamabe et al. [15] investigated the distribution of *M. oralis* in Japanese patients with periodontitis and examined the serum IgG responses to archaeal components. Western immunoblotting detected IgG antibodies against *M. oralis* in sera from 8 of 11 tested patients suggesting the potential of *M. oralis* as an antigenic component of periodontitis. Furthermore, in a follow-up study Yamabe et al. [16] identified one of the antigenic molecules as subunits of the type II chaperones (Cpn II = heat shock proteins that occur in *Archaea* and *Eukarya*). The authors [16] also demonstrated cross-reactivity with the human chaperonin CCT applying western immunoblotting. This is especially important given our knowledge regarding cross-reactivity of bacterial heat shock proteins with human molecules. For instance the bacterial Hsp60 (group I Cpn) are known to be highly antigenic molecules [17, 18] present in many pathogenic bacteria, and several immune disorders, such as rheumatoid arthritis or rheumatoid fever, are thought to be triggered by these molecules. Although further investigation is needed for definite conclusions, the data indicate that antigenic molecules of *M. oralis* have the potential to act as modifier or even initiator of inflammation in periodontal lesions.

## 4. Methanogens in the Human Gut System: What Is New?

As we know by the results of virtually all studies *M. smithii* is the major archaeal component in the human gut system, while *Methanosphaera stadtmanae* is a less frequently detected species. The importance of *M. smithii* in this complex microbial ecosystem, primarily its role as H_2_-consumer and supporter of the fermenting microbiota, has been previously addressed [6, 7]. H_2_-consumption and data regarding interactions among the three hydrogenotrophic microbial groups in the gut and their possible relation to disease have been discussed more comprehensively recently. The disorders in which methanogens are probably involved are inflammatory bowel disease (or Crohn's disease), irritable bowel syndrome, colorectal cancer, diverticulosis, and obesity. For detailed information the reader is referred to the articles by Nakamura et al. [8] and Roccarina et al. [19]. In the essence, a direct or indirect contribution of methanogens to the development of gastrointestinal disorders is unclear and remains an ongoing subject of debate. Currently it is impossible to draw definite conclusions because of fragmentized and even controversial findings. In particular, little is known regarding the extent to which the hydrogenotrophic microbiota varies in composition or metabolic specificity among individuals and how the interactions with the remaining microflora affect our health. By way of example, let us consider the findings by Abell et al. [20]. The authors found a negative correlation between mean fecal butyrate concentration and methanogen abundance by testing 8 individuals weekly over a 12-week period by using molecular methods. Their data suggest an indirect association of methanogens with colorectal cancer or other gastrointestinal disorders, considering the recognized importance of butyrate as a primary energy source for cells lining the colon and for protecting against activities associated with carcinogenesis (e.g., enhancement of cell cycle arrest and apoptosis, [21, 22]). However, it is unclear what the underlying mechanisms are that link high numbers of methanogens with low butyrate concentrations and what are the cause and effect. One explanation could be that methanogens live in syntrophy with butyrate degrading organisms via interspecies H_2_-transfer. While such interactions exist in other environments, to our knowledge respective syntrophic bacterial partners (i.e., members of the family *Syntrophomonadacaea*) are not in the molecular inventories of the microbial gut environments [1]. Abell et al. [20] speculated instead that in the presence of high numbers of methanogens butyrate could not be formed by known gut bacteria using acetate, such as *Faecalibacterium prausnitzii* and *Roseburia intestinalis*, because acetate-producing organisms (acetogens) had been outcompeted by the methanogens. This scenario would imply that increased methanogenic activity in the gut leads to reduced availability or concentrations of acetate. But this in turn conflicts with the results of Samuel and Gordon [23], which suggest that syntrophic archaeal-bacterial interactions in the gut lead to increased acetate levels. From these examples it becomes clear that more studies are needed for drawing definite conclusions and that the identity of syntrophic partners is crucial here, as they modulate the type and concentration of short-chain fatty acids in the gut system. The latter study is also interesting with respect to the human physiological disorder obesity. Based on a gnotobiotic mouse model a link between *M. smithii* and host energy balance could be established [23]. The colon of germ-free mice was colonized with a polysaccharide-degrading bacterium (*Bacteroides thetaiotaomicron*), a sulfate-reducing bacterium (*Desulfovibrio piger*), and *M. smithii* in different combinations [23]. *B. thetaiotaomicron* degraded polyfructose-containing glycans more efficiently in the presence of *M. smithii*. In addition, mice colonized with these two microbes showed increased fermentation products and an increase in adiposity (increased storage of energy in fat cells). These effects were not observed after cocolonization with the pair *B. thetaiotaomicron* and *D. piger* (as alternative H_2_-consumer), emphasizing the critical role of *M. smithii* in promoting polysaccharide degradation, followed by absorption of the fatty acids that lead to liver lipogenesis and formation of fat deposits. These experiments substantiate the hypothesis, that, by providing the final step in energy extraction from degradation of organic compounds, methanogens could alter the whole gut physiology with profound consequences for the host. As such they could also be an interesting target for therapeutical manipulation of obesity. In fact, further evidence has recently been provided by Zhang et al. [24]. Using real-time PCR, they detected significantly higher numbers of H_2_-utilizing methanogenic *Archaea* in three obese individuals than in three normal-weight or three post-gastric-bypass individuals. The numbers of the H_2_-producing *Prevotellaceae* were also highly enriched in the obese individuals supporting the hypothesis that interspecies H_2_-transfer between bacterial and archaeal species is an important mechanism for increasing energy uptake by the human large intestine in obese persons. The speculated interactions between methanogens and *Prevotellaceae* however need further verification because of the low number of individuals tested. 

The above examples [23, 24] were chosen here, because somewhat conflicting results have been obtained by Armougom et al. [25] who found the number of *M. smithii* in 20 obese persons compared to 20 normal weight persons not significantly increased. Instead they found a significantly increased number of *M. smithii* in nine anorexia nervosa patients compared to the normal weight persons. This result, also produced by real-time quantitative PCR, shows that more research is needed to truly understand the ecology of the human gut flora and the role of archaea for intestinal disorders.

## 5. An Update on *Euryarchaeota* Diversity in Humans

*M. smithii*, *M. oralis* as well as *Methanosphaera stadtmanae*, are the only archaeal species that have been successfully cultivated and isolated from human habitats (i.e., the intestinal tract, vagina, and oral cavity) in the past. Applying PCR-based technologies targeting the 16S rRNA gene and/or the *mcrA* gene directly to clinical specimens has confirmed the presence of these species in relevant numbers in several studies. Nonetheless recent findings have shown that the diversity of *Archaea* in humans is higher and that additional taxa, that have been recovered by molecular methods, all belong to the group of *Euryarchaeota*. 

As a close relative to *M. smithii* and *M. oralis*, we have previously verified the existence of a third phylotype (“phylotype 3”) in periodontal pockets and within infected dental root canals [26]. First 16S rRNA gene sequences of this putative organism had already been reported earlier (e.g., [27]), however only our combined recovery of both 16S rRNA and *mcrA* gene sequences with fitting topology of inferred phylogenetic trees from the same individual gave sufficient confidence that humans are colonized by a third *Methanobrevibacter* phylotype. This finding may sound trivial given the high archaeal diversity in environmental habitats, but not given the extreme reduced archaeal diversity in humans. Interesting questions that arise are, whether this uncultured organism shares individual niches or competes with *M. oralis* and why it has escaped cultivation so far. Though it is likely that this organism is cultivable with the same conditions as *M. smithii* and *M. oralis*, failure so far is probably due to its lower prevalence in human clinical samples [26]. 

Irrespective of this, the diversity of human-associated *Euryarchaeota* in the gut system seems to be larger than previously thought according to two independent studies. Scanlan et al. [28] and Mihajlovski et al. [29] reported the identification of *mcrA *gene sequences only distantly related to cultured methanogens from the five recognized orders (i.e., *Methanomicrobiales*, *Methanopyrales*, *Methanobacteriales*, *Methanococcales*, and *Methanosarcinales*). It has been hypothesized that this novel *mcrA* gene type corresponds to an uncultured phylotype that makes up a putative sixth methanogenic order [29]. In a subsequent extended study, Mihajlovski et al. [30] identified four additional *mcrA* gene types also grouping within the same clade of this putative sixth methanogenic order. In addition, these sequence types shared close to moderate similarity with a number of clone sequences recovered from animal studies such as pig feces and from cattle rumens [30], further supporting the possibility of a novel group of *Euryarchaeota* adapted to the animal intestinal tract. By comparing fecal samples from human volunteers of different ages Mihajlovski et al. [30] also found that these novel sequence types were significantly more prevalent in elderly people than in young people. Interestingly the same samples containing the novel *mcrA* sequence types also revealed 16S rRNA gene sequences grouping within the *Thermoplasmatales* but with no cultured relatives. If corresponding to each other, the conclusion would be that a novel sister group of *Thermoplasmatales* probably capable of methanogenesis exists in the human gut system. Otherwise, one could conclude that the *mcrA*-genotypes may represent organisms that share the gut environment with other archaeal species related to *Thermoplasmatales*. *Thermoplasma*-like sequences have also been identified in the periodontal pockets [31], however, with a sequence identity to those sequences found in the human gut of only 93%, probably reflecting specific adaptation to this particular human ecosystem. In any case these findings emphasize the increased diversity of *Euryarchaeota* in humans, in both the intestinal tract and the oral cavity. 

Further novel insights regarding archaeal diversity in humans were previously reported by Oxley et al. [32]. In an analysis of the microbiota in colonic mucosal biopsies from 8 out of 39 patients with inflammatory bowel disease they found 16S rDNA sequences representing a phylogenetically rich diversity of halophilic archaea (15 different phylotypes) from the *Halobacteriaceae *(haloarchaea), including those with no directly related cultured representatives. Furthermore, aerobic enrichment cultures prepared from a patient biopsy at low salinity (2.5% NaCl) yielded haloarchaea sequence types. Microscopic observation after fluorescence *in situ* hybridization provided evidence of the presence of viable archaeal cells in these cultures. These results prove the survival of haloarchaea in the human digestive system and suggest that they may be members of the mucosal microbiota. Whether they constitute regular colonizers and whether they occur in abundance comparative to methanogenic archaea are unknown so far. The study of Oxley et al. [32] is the first that clearly shows that the diversity of viable *Euryarchaeota* in humans goes beyond methanogenic archaea. What the relationship of the occurrence of halophilic archaea is with respect to inflammatory bowel disease remains speculative for now. One possibility is that they stem from precolonoscopy saline lavage solutions. However, Oxley et al. [32] consider this possibility as unlikely, since they detected different halophilic phylotypes in saline lavage solutions not directly related to those found in the biopsies. This aspect by its own is puzzling, as it indicates that the manufacturer providing precolonoscopy saline lavage solutions used salt from natural sources directly without sterilisation. Alternatively the solution itself may contain only the DNA of killed cells, left after heat-sterilisation and/or filtration. In any case, Oxley et al. [32] found also halophilic archaea in some patients that had not received the saline lavage solution. In addition halophilic archaea have also been reported in fecal samples from Korean probands, who were not suffering from Crohn's disease [33], leaving the question for the origin of those extremophilic archaea unanswered for now. Since the human colon is not an obvious salty environment, the physiological basis of an opportunistic colonization by haloarchaea as indicated by the above studies is another intriguing question.

In summary, recent findings have enlarged the known diversity of euryarchaeota in humans, now including further phylotypes of *Methanobrevibacter*, but even an entire novel order of methanogens, members of *Thermoplasmatales,* and *Halobacteriac*. The question arises here, why their presence has largely eluded detection in many studies before. One apparent answer is that the primers, used to amplify their DNA, were not specific and/or sensitive enough. From our experiences, most primers designed in older studies for characterization of environmental archaea are not suitable for detection of human-associated archaea. In many cases cross-reaction with human DNA was observed in our laboratory, sometimes even leading to PCR bands with the size indicating successful archaeal 16S rRNA gene amplification. Yet, even more frustrating were the results from subsequent sequence analysis. Hence, careful design of novel primers as well as the use of multiple molecular targets (16S rDNA and *mcrA*) is highly important in recovering a wider range of human methanogens. An alternative approach may be the separation of human and microbial DNA prior to PCR amplification. Two different approaches have been developed [34], which might also be useful for metagenomic studies of the human microbiome [35]. The fact that the choice of the molecular approach greatly influences our perception of archaeal diversity and prevalence in humans can be best illustrated in another most recent study. Dridi et al. [36] developed a new protocol for the extraction and PCR-based detection of *M. smithii* and *M. stadtmanae* DNA in human stool samples. The protocol included a mechanical lysis step with glass beads which was applied twice combined with an overnight incubation with proteinase K. PCR-based detection included newly designed primers targeting the 16S rRNA gene but also the *rpoB* gene, which encodes the *β* subunit of RNA polymerase, one of the core genes shared by *Bacteria* and *Archaea. *By testing fecal samples from 700 volunteers by RTQ-PCR, they found *M. smithii* in virtual all individuals and *M. stadtmanae* in almost 30% of cases. Double treatment with glass beads was performed because gut methanogens have been shown to possess a proteinase K resistant cell wall [37]—and apparently the extensive mechanical action was decisive in the efficiency of DNA extraction. Similarly Salonen et al. [38] found an increased prevalence of methanogenic *Archaea* in human fecal samples by repeated mechanical disruption steps, compared to other extraction protocols. These findings revise our perspective that methanogens colonize the gut of only about half of the human population. Instead, *M. smithii* appears as an almost ubiquitous inhabitant of the intestinal microbiome. As such it sheds additional light on the paramount role that methanogenic species may have in the overall microbial ecology of the digestion process. For deeper understanding it is therefore even more important to monitor changes in the archaeal gut flora under different physiological, pathological, and therapeutical (e.g., administration of antibiotics) conditions.

## 6. Open Questions

### 6.1. Have We Already Grasped the Entire Archaeal Diversity in Humans, and Is This Diversity Really Confined to the Group *Euryarchaeota*?

With the use of redefined primer systems and application of modern molecular techniques such as deep sequence analysis via pyrosequencing and with the expansion to other habitats (e.g., the human stomach, the oesophagus), it is likely that some surprising findings regarding archaeal prevalence and diversity will show up in the near future. Whether or not archaea other than euryarchaeota are colonizers of the human body is unknown so far. However, members belonging to *Crenarchaeota* have been detected in the human gut system previously with PCR-based methods [39]. In order to reproduce this intriguing result we tested the same primers and PCR conditions in our laboratory. No 16S rRNA genes of crenarchaeota were obtained in our study, neither from five selected fecal nor from five oral samples, so that the findings of Rieu-Lesme et al. [39] remain anecdotal for now. In addition, when testing further *Crenarchaeota* specific-primers [40] we only amplified and detected the 16S rRNA gene of *M. oralis* in oral samples, indicating an apparent lack of crenarchaeota and an abundance of *M. oralis* sufficiently high enough to overcome mismatches to the crenarchaeota primers. In another first attempt we tested published primers specifically designed for the detection of *Korarchaeota* [41] and *Nanoarchaeota* [42] with oral samples. In both cases we observed cross-reaction with human DNA, even under highly stringent PCR conditions. Again, new primer systems and/or efficient strategies for removal of human DNA are required to find clear answers.

### 6.2. What Prevents Most Archaea from Colonization of the Human Body?

Apparently, the human microbial ecosystem follows the trend of many environmental habitats, in which the observed diversity of bacteria is higher than that of archaea [43]. The underlying mechanisms for this, though unknown, may be partially similar. However, within humans additional selective forces such as the immune system affect the microbial colonization pattern. And, once established, the human physiological microflora seems to defend its own acquired status very effectively against “newcomers.” This may even, at least partially, explain the differential colonization pattern of the oral and gut environment [44]. One could also speculate that some archaea simply do not have the opportunity to colonize the human body but once they get access colonization is possible. The presence of *Halobacteriaceae* in Morbus Crohn patients, probably inserted through contaminated salt lavage solution, may be an example for this [32]. Conversely, several viable methanogenic taxa (others than those so far detected in humans) can be found in food, primarily in vegetables but also in nuts and meat [45]. In addition, nonmethanogenic archaea, including crenarchaeota, have been found in fermented seafood [46]. Hence, those organisms (with the possible exception of halophilic archaea) get the opportunity, however, seem incapable for steady colonization. One possible explanation that most archaea cannot colonize the human body could be the uniqueness of their biochemistry. *Archaea* use a variety of “exotic” cofactors that bacteria or eukaryotes neither synthesize nor require [47]. So, from the standpoint of nourishment human ecological niches could be an unpleasant environment for archaea, as they are as “biochemical outsiders” in an inferior position when competing with bacteria. Even more exciting then becomes the question, which strategies *M. smithii* and *M. orali*s and other human archaea have developed to overcome this key disadvantage. Whole genome analysis of several *M. smithii* strains in comparison with close relatives from environmental systems has shown, that *M. smithii* is in fact highly adapted to the gut system [48]. This includes its capability of (i) production of surface glycans resembling those found in the gut mucosa, (ii) expression of adhesion-like proteins, (iii) consumption of a high variety of fermentation products by saccharolytic bacteria, and (iv) effective competition for nitrogenous nutrient sources [48]. It will be highly interesting to compare its genome with that of *M. oralis*, discovering which genes are responsible for successful colonization of the human oral cavity versus gastrointestinal tract.

### 6.3. Which Are the Syntrophic Partners of Methanogens in Humans, and Are These Organisms Consistent or Vary over Time and Space and among Individuals?

Possible bacterial candidates can be deduced from correlation analysis of species prevalence and abundance using molecular methods. For instance, some evidence exists for possible interactions between H_2_-producing *Prevotellacea* and *M. smithii* in the human gut system [24] or *Synergistes* and *M. oralis* in endodontic infections [26, 49]. A manuscript from our laboratory is currently underway describing the correlations between *M. oralis* and recognized periodontal pathogens. Such correlations, however, not necessarily prove syntrophic interactions but can also mean that other unknown factors favour the simultaneous growth of both archaeal and bacterial niche partners. More precisely, it can be concluded that *Bacteroides thetaiotaomicron* and *M. smithii* can cooperatively degrade polysaccharides, as the gnotobiotic mice studies of Samuel and Gordon [23] have shown. While it is plausible to assume that this interaction truly occurs in the human gut, there might exist a larger spectrum of syntrophic partners. Stable isotope probing (SIP) of different organic compounds, such as butyrate or propionate has become a popular method to investigate syntrophic methanogen-bacterial interactions in various habitats (e.g., [50, 51]). In order to understand the full range of possible syntrophic partners in humans, analogous experiments could be designed based on the incubation of human fecal or oral plaque samples with various short-chain fatty acids labelled with ^13^C followed by subsequent analysis of marker genes including those involved in methanogenesis (e.g., the *mcrA*). Such experiments would not only lead to the identification of interacting bacterial partners but also—once established—enable to monitor the dynamics of such partnerships over time through repeated sampling and testing. For example, the feasibility of linking structure and function of human microbes using SIP has recently been demonstrated in a study analyzing denitrification in human dental plaque samples [52]. 

### 6.4. How likely Is the Involvement of Archaea in Infectious Processes of Otherwise Sterile Sites Such as Brain Abscesses, Peritonitis, or Endocarditis?

This is an intriguing question, given that distinct members of the endogenous microflora (from the oral cavity or gut system), while harmless in their natural location, can cause severe life-threatening infections. Syntrophic interactions with methanogens could be a driving factor for such kind of polymicrobial diseases, provided that methanogens also get access to primary sterile sites. One already proven example is the infection of a dental root canal leading to apical periodontitis [12–14]. However, our initial attempts to look for methanogens in extraoral, “real” clinical samples were unsuccessful so far, but most likely due to the dominating amounts of human DNA cross-reacting with the primer systems. The newly designed primer systems mentioned above as well as more efficient DNA extraction methods may help to give clarity here. However, if methanogenic archaea or nonmethanogenic archaea play a role in life-threatening infections, their sheer diagnosis on the sole base of molecular studies would not be sufficient. Given the great implication of such findings (e.g., resistance to most antibiotics and need for entire new targets for therapy or prevention), it would be of utmost importance to obtain cultured isolates for further verification and phenotype characterization including resistance/susceptibility testing. 

## 7. Final Remarks

Archaeal colonization of human anatomical sites has long been neglected as research objective. Conversely, there has been quite some attention for archaea and their role in animals, including but not limited to ruminants and insects (reviewed in [53]). It is possible that archaeal interactions with the animal host parallel the situation in humans. In particular termites have been relatively well studied, and interestingly the hindgut of so-called lower termites harbours mainly archaeal species closely related to *Methanobrevibacter* [54]. Termites might therefore function as a suitable and easy to study model ecosystem for gaining more insights into the biological basis of archaeal colonization in humans.

A major step toward a better understanding of the function and dynamics of human archaea will be done by results originating from the Human Microbiome Project [35]. Given the recognized plasticity of the genomes of most opportunistic pathogenic bacteria (e.g., acquisition of virulence factors or antibiotic resistance mechanisms), it is important to assess the genomic architecture of *M. smithii* and *M. oralis* in different individuals as well. Metagenomic analyses for establishing a microbial gene catalogue of the human gut or oral cavity [55] will not only reveal whether or not horizontal gene transfer is also common among those archaea but also—once a sufficiently high number of individuals are tested—if certain human physiological disorders are potentially linked with a given archaeal strain (or phylotype). In addition, concerted activities of clinicians and microbiologists from different areas (i.e., medical and archaea experts) will be needed in future to elucidate (and possibly interfere with) the role of *Euryarchaeota* in human health and disease.
